# Post–COVID-19 Expansion of *Streptococcus pyogenes* Lineages Causing Toxic Shock Syndrome, Japan, 2023–2024

**DOI:** 10.3201/eid3211.260336

**Published:** 2026-11

**Authors:** Tadayoshi Ikebe, Hitoshi Otsuka, Mari Sasaki, Takahiro Yamaguchi, Yoshimi Date, Yu Kazawa, Kazuki Saito, Kaho Ikeda, Rumi Okuno, Yukihiro Akeda, The Working Group for Beta-Hemolytic Streptococci in Japan

**Affiliations:** National Institute of Infectious Diseases, Tokyo, Japan (T. Ikebe, Y. Akeda); Yamaguchi Prefectural Institute of Public Health and Environment, Yamaguchi, Japan (H. Otsuka); Oita Prefectural Institute of Health and Environment, Oita, Japan (M. Sasaki); Osaka Institute of Public Health, Osaka, Japan (T. Yamaguchi); Kanagawa Prefectural Institute of Public Health, Kanagawa, Japan (Y. Date); Fukushima Prefectural Institute of Public Health, Fukushima, Japan (Y. Kazawa); Toyama Institute of Health, Toyama, Japan (K. Saito, K. Ikeda); Tokyo Metropolitan Institute of Public Health, Tokyo (R. Okuno)

**Keywords:** Streptococcus pyogenes, streptococcal toxic shock syndrome, bacteria, M1_UK_, M1_13SNPs_, *emm1*, COVID-19, Japan, molecular epidemiology, SARS-CoV-2, severe acute respiratory syndrome coronavirus 2, viruses, respiratory infections, zoonoses, Japan

## Abstract

We analyzed 919 *Streptococcus pyogenes* isolates from laboratory-confirmed streptococcal toxic shock syndrome cases in Japan during 2023–2024. Post–COVID-19 resurgence was driven by expansion of M1_UK_ (49.8%) and emerging M1_13SNPs_ (37.4%) lineages, replacing ancestral M1_global_. M1_13SNPs_ were associated with younger patient age and higher death rates in unadjusted analyses.

Streptococcal toxic shock syndrome (STSS) is a rapidly progressive, life-threatening invasive disease primarily caused by group A *Streptococcus* (*Streptococcus pyogenes*) bacteria (*1*,*2*). After relaxation of nonpharmaceutical interventions for COVID-19 in Japan, resurgence in STSS was observed, coinciding with reports of M1_UK_ lineage expansion (*3*). We conducted a nationwide molecular epidemiologic study to clarify the contribution of *emm1* sublineages to this increase.

## The Study

This laboratory-based study analyzed *S. pyogenes* isolates from laboratory-confirmed STSS cases reported across Japan during January 1, 2023–December 31, 2024. STSS is notifiable under Infectious Disease Control Law; surveillance is coordinated by the National Institute of Infectious Diseases (NIID; Tokyo, Japan) with prefectural public health institutes and sentinel hospitals. We applied US Centers for Disease Control and Prevention criteria: isolation of group A *Streptococcus* from a normally sterile site plus clinical shock with multiorgan involvement (*4*).

A total of 919 isolates were collected from 1,477 reported STSS cases, 186 isolates in 2023 and 733 in 2024. Coverage was 62.2% overall, 45.9% in 2023, and 68.4% in 2024. Specimens from routine care were submitted to 7 regional reference laboratories (Fukushima, Tokyo, Kanagawa, Toyama, Osaka, Yamaguchi, and Oita) and NIID. Species identification was performed using API 20 Strep (bioMérieux, https://www.biomerieux.com), and Lancefield group determination used latex agglutination kits (Iwaki Corporation, https://www.iwaki-kk.co.jp). The NIID Ethics Review Board approved this public health surveillance under Article 15 of the Infectious Disease Law; individual consent was waived.

We performed *emm* genotyping by PCR and sequencing of the 5′ region using primers emm-1/emm-2 and sequencing primer emmseq2 with 180-bp sequences matched to the CDC emm database (*5*). We assessed *speA* and *speC* genes by primers described previously (*6*). Among the *emm1* isolates, we identified M1_UK_, M1_23SNPs_, M1_13SNPs_, and M1_global_ lineages using allele-specific PCR targeting single-nucleotide polymorphisms (SNPs) in *rofA*, *gldA*, and *pstB* (*7*). 

To investigate the differences between the M1_UK_ strain and the M1_13SNPs_ strain, we amplified the upstream region of the *speA* gene using the M1UK forward (cctagtctatacagccaaaaagc) primer and the M1UK reverse (gcattgatggagctggtggg) primer and sequenced them using the M1UKseq1 (tatacagccaaaaagcgagg) primer. We confirmed that mutations observed in the M1_UK_ strain were not present in all the strains obtained as M1_13SNPs_, which was consistent with the results of allele-specific PCR targeting SNPs in *rofA*, *gldA*, and *ptsB*. We conducted statistical analyses in Ekuseru-Toukei version 4.10 (Social Survey Research Information Co., Ltd, https://bellcurve.jp) using *t* test for age and Fisher exact test for deaths; we considered p<0.05 significant.

Among 919 STSS isolates, we identified 31 *emm* types, including 2 untypeable strains with signal sequence deletions (Appendix Table 1, https://wwwnc.cdc.gov/EID/article/32/11/26-0336-App1.pdf). Type *emm1* predominated (470/919, 51.1%), followed by *emm12* (12.3%), *emm89* (11.5%), *emm49* (7.9%), and *emm81* (5.8%), accounting for >85% of isolates overall (Appendix Figure 1, panel A). After adjusting for collection rates (Appendix Table 2), e*mm1* increased markedly in 2024 (Appendix Figure 1, panel B). National surveillance data showed *emm1*-associated STSS declined during 2020–2022 but rose after mask policy relaxation (March 13, 2023), reaching 60–80 cases/month during January–June 2024 (*8*,*9*). Most other types (*emm89*, *emm81*, *emm49*) remained stable, whereas *emm12* also increased (Figure 1).

**Table 1:** Clinical outcomes by lineage in study of post–COVID-19 expansion of *Streptococcus pyogenes* causing toxic shock syndrome, Japan, 2023–2024*

Lineage	No. cases	Average age (IQR), y	p value†	No. (%) deaths	p value†
M1_UK_	234	54.6 (41–69)	<0.0001	48 (20.5)	0.4403
M1_13SNPs_	176	58.5 (43–75)	0.0391	55 (31.3)	0.0412
M1_global_	60	58.9 (46–72)	0.1158	12 (20.0)	0.7431
Non-*emm1*	449	62.7 (50–78)	Referent	104 (23.2)	Referent

*IQR, interquartile range. †By Fisher exact test, significant at p<0.05 comparison with non-*emm1.* p values are unadjusted and should be interpreted with caution because they do not account for potential confounding factors such as underlying conditions, disease severity, or differences in initial treatment.

**Figure 1:**
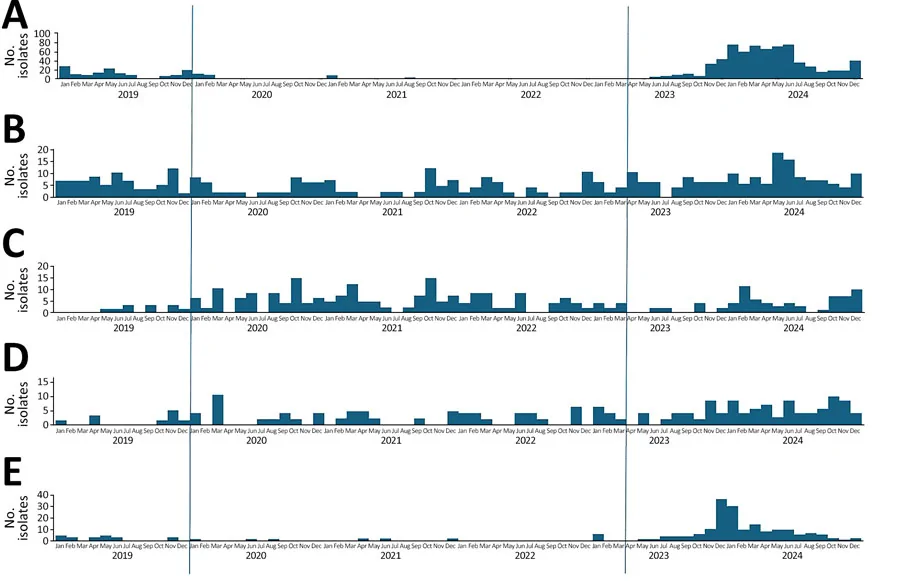
Isolates of *emm* gene types by month in study of post–COVID-19 expansion of *Streptococcus pyogenes* lineages causing toxic shock syndrome, Japan, 2023–2024. y-axis indicates the number of calculated 100% isolates of each *emm* type: A) *emm1*; B) *emm89*; C) *emm81*; D) *emm49*; E) *emm12*. Vertical lines separate pre–COVID-19, COVID-19, and post–COVID-19 periods.

**Table 2:** Characteristics of patients with toxic shock syndrome by the causative strain of *Streptococcus pyogenes*, Japan, 2023–2024*

Characteristic	No. (%) M1_13SNPs_	No. (%) with non-*emm1*	Crude RR (95% CI)	Crude OR (95% CI)	p value†
No. patients	176	449			
Sex					
M	94 (53.4)	273 (60.8)	Referent	Referent	
F	82 (46.6)	176 (39.2)	1.09 (0.98–1.21)	1.35 (0.95–1.92)	0.1040
Age group, y					
0–24	12 (6.8)	17 (3.8)	1.03 (0.99–1.08)	1.86 (0.87–3.98)	0.1368
25–39	22 (12.5)	39 (8.7)	1.04 (0.98–1.11)	1.50 (0.86–2.61)	0.1768
40–54	37 (21.0)	83 (18.5)	1.03 (0.85–1.13)	1.17 (0.76–1.81)	0.4985
55–69	43 (24.4)	118 (26.3)	0.98 (1.88–1.08)	0.91 (0.61–1.36)	0.6847
70–84	48 (27.3)	134 (29.8)	0.96 (0.87–1.08)	0.88 (0.60–1.30)	0.5579
>85	14 (8.0)	58 (12.9)	0.85 (0.89–1.00)	0.58 (0.32–1.07)	0.0944
Deaths					
All ages	55 (31.3)	104 (23.2)	1.12 (1.00–1.25)	1.51 (1.02–2.22)	0.0412
0–24	0 (0)	1 (5.9)	0.94 (0.84–1.06)	0	1.0000
25–39	8 (36.4)	4 (10.3)	1.41 (1.01–1.97)	5.00 (1.29–19.3)	0.0202
40–54	4 (10.8)	19 (22.9)	0.86 (0.74–1.02)	0.41 (0.13–1.30)	0.1395
55–69	13 (30.2)	22 (18.6)	1.17 (0.94–1.44)	1.89 (0.85–4.20)	0.1326
70–84	23 (47.9)	39 (29.1)	1.36 (1.01–1.82)	2.24 (1.14–4.41)	0.0217
>85	7 (50.0)	19 (32.8)	1.34 (0.77–2.33)	2.05 (0.63–6.70)	0.3525

*RR and OR between M1_13SNPs_ and non-*emm1* are derived from univariate logistic regression analysis. OR and p values are unadjusted and should be interpreted with caution because they do not account for potential confounding factors such as underlying comorbidities, disease severity, or differences in initial treatment. OR, odds ratio; RR, risk ratio. †By Fisher exact test.

Among 470 *emm1* isolates, we identified M1_UK_ (234 [49.8%]), M1_13SNPs_ (176 [37.4%]), and M1_global_ (60 [12.8%]) lineages. Compared with 2019 (M1_global_ >60%; M1_UK_ 24%; M1_13SNPs_ 12%), 2023–2024 showed replacement: M1_UK_ increased from 44% to 51% and M1_13SNPs_ from 29% to 39%, but M1_global_ decreased from 26% to 11% (Appendix Figure 2). Each lineage rose after mask relaxation (Figure 2).

**Figure 2:**
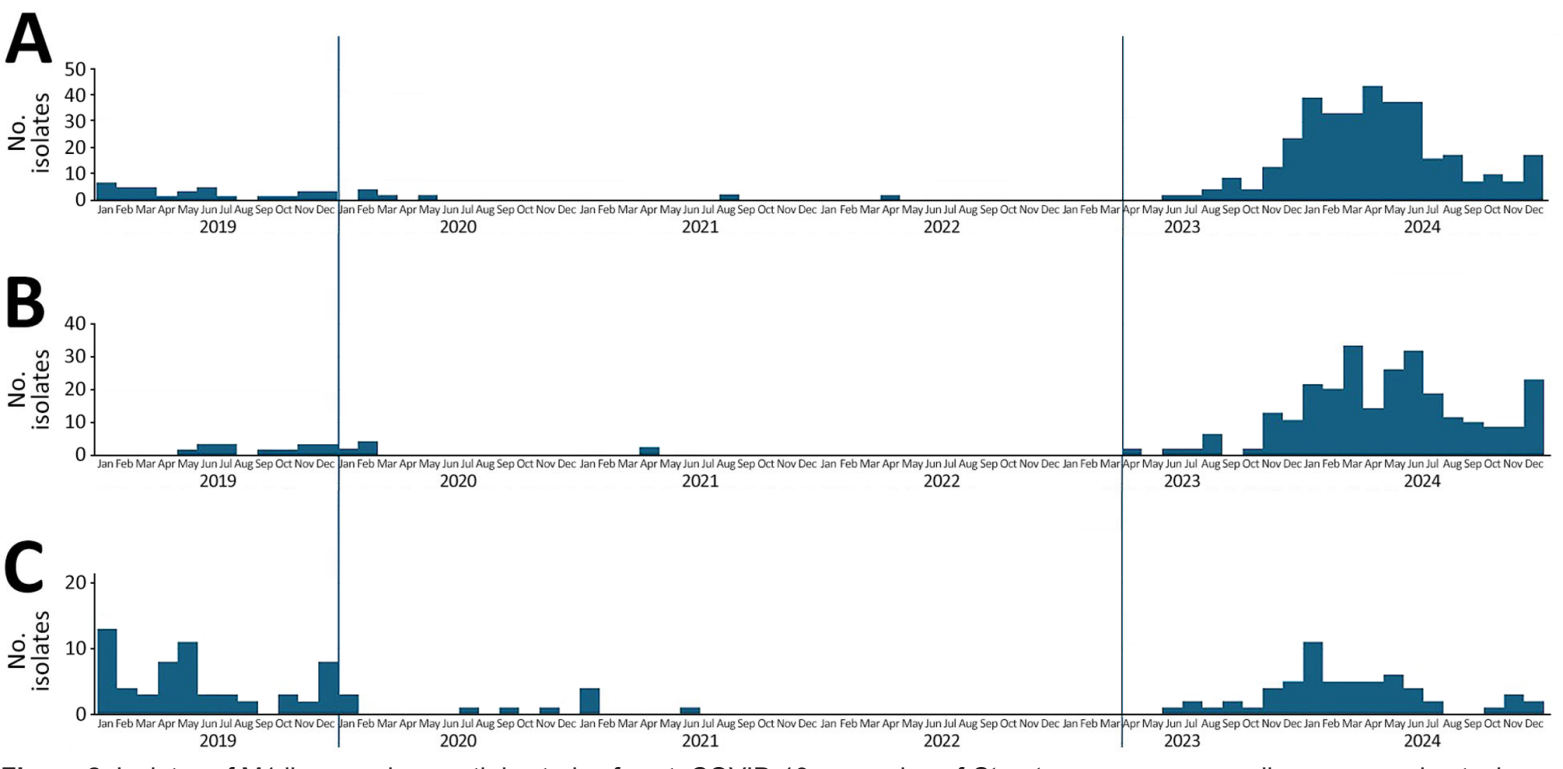
Isolates of M1 lineages by month in study of post–COVID-19 expansion of *Streptococcus pyogenes* lineages causing toxic shock syndrome, Japan, 2023–2024. y-axis indicates the number of calculated 100% isolates of each lineage: A) M1_UK_; B) M1_13SNPs_; C) M1_global_. Vertical lines separate pre–COVID-19, COVID-19, and post–COVID-19 periods.

Among the 470 *emm1* isolates, 458 (97.4%) were *speA* positive: 283 (60.2%) *speA*-only, 175 (37.2%) both *speA* and *speC*, and 8 (1.7%), *speC*-only. Four (0.9 %) were positive but for neither *speA* nor *speC*. M1_UK_ mainly carried *speA*-only (202 [86.3%]), M1_13SNPs_ harbored both genes (143 [81.3%]), and M1_global_ was predominantly *speA*-only (55 [90.2%]) (Appendix Table 3).

M1_13SNPs_ infections occurred in younger patients (average age 58.3 years; interquartile range [IQR] 43–75 years) and were associated with higher risk for death (mortality rate 31.3%) than non-*emm1* cases isolated during the same period, in which the average age was 62.7 (IQR 50–78) years and mortality rate 23.2%. M1_UK_ also affected younger patients (average age 54.9 [IQR 41–69] years) but showed comparable mortality rate (20.5%), whereas M1_global_ showed no significant differences in either age (average age 58.9 [IQR 46–72] years) or mortality rate (20.0%) (Table 1).

M1_13SNPs_ infections are associated with younger patients and higher mortality rate. Because age is a major determinant of STSS outcomes, we assessed the association between age and mortality to confirm that the observed differences in mortality were not influenced by age. Although we observed no significant differences in sex or age distribution between cases of STSS caused by M1_13SNPs_ and those caused non-*emm1* strains (Table 2), mortality was significantly higher in patients with M1_13SNPs_ infections (31.3% vs. 23.2%; odds ratio [OR] 1.51 [95% CI 1.02–2.22]). Age-stratified analysis further revealed significantly higher mortality in the M1_13SNPs_ group among patients 25–39 years of age (OR 5.00 [95% CI 1.29–19.3]) and 70–84 years of age (OR 2.24 [95% CI 1.14–4.41]).

## Conclusions

Nationwide surveillance links Japan’s post–COVID-19 STSS resurgence to expansion of *emm1 S. pyogenes*, driven by replacement of M1_global_ with M1_UK_ and M1_13SNPs_ lineages. That pattern parallels M1_UK_ increases reported in Europe (*9*,*10*) and highlights the emergence of M1_13SNPs_, which was associated with higher mortality than were non-*emm1* strains, as well as diversity in *speA/speC* profiles and a younger age distribution.

The temporal association with relaxation of nonpharmaceutical interventions suggests increased opportunities for transmission in school and community settings. Clinical heterogeneity observed among *emm1* sublineages underscores the value of lineage-level molecular surveillance for risk assessment.

Limitations of our study included 62% coverage of reported cases, restriction to STSS cases, limited study periods, and incomplete clinical data use that required us to perform unadjusted analyses. Although infection with M1_13SNPs_ was associated with higher mortality, that finding should be interpreted cautiously because it might be affected by unmeasured confounding factors such as underlying conditions, treatment differences, or disease severity. Our results should therefore be considered exploratory and do not establish an independent effect of the M1_13SNPs_ lineage on clinical outcomes.

Nonetheless, application of standardized national methods to 919 isolates provided evidence of rapid lineage replacement. We recommend continued molecular surveillance needed to monitor M1_UK_ and M1_13SNPs_ globally, identify emerging variants, and inform public health responses to future *S. pyogenes* epidemics.

Appendix Additional information about post–COVID-19 expansion of *Streptococcus pyogenes* lineages causing toxic shock syndrome, Japan, 2023–2024.
