# Supplementary material for: Post–COVID-19 Expansion of Streptococcus pyogenes Lineages Causing Toxic Shock Syndrome, Japan, 2023–2024
Source: Emerg Infect Dis. 2026 Nov;32(11):1902–6. doi: 10.3201/eid3211.260336 (PMC13715874; doi:10.3201/eid3211.260336)
Supplement: Supplementary file 1 [file 26-0336-Techapp-s1.pdf]

Article DOI: https://doi.org/10.3201/eid3211.260336

EID cannot ensure accessibility for supplementary materials supplied by authors. Readers who have difficulty accessing supplementary content should contact the authors for assistance.

Post–COVID-19 Expansion of *Streptococcus pyogenes* Lineages Causing Toxic Shock Syndrome, Japan, 2023–2024

Appendix

Appendix Table 1. Distribution of *emm* types among 919 *Streptococcus pyogenes* isolates for streptococcal toxic shock syndrome (STSS), Japan, 2023–2024. Two isolates were untypable because of signal sequence deletions.

emm-type	2023	2024	Total
1	61	409	470
3	0	1	1
4	0	22	22
6	0	1	1
9	1	0	1
11	3	8	11
12	36	77	113
22	2	0	2
25	0	1	1
28	1	1	2
33	0	1	1
44	2	0	2
49	21	52	73
58	0	2	2
73	1	0	1
75	1	1	2
76	1	2	3
77	4	13	17
81	9	44	53
82	0	3	3
83	0	1	1
87	3	7	10
89	32	74	106
90	1	1	2
91	1	0	1
98	1	1	2
104	0	2	2
106	2	3	5
108	1	4	5
112	1	0	1
221	0	1	1
Untypable*	1	1	2
Total	186	733	919

*Signal sequence deletion.

Appendix Table 2. The collection rate of strains from year to year in STSS isolates in Japan, 2023–2024

Characteristic	2019	2020	2021	2022	2023	2024	Total	2023–2024 Period
Number of cases	411	269	177	209	405	1,072	2,543	1,477
Number of isolates	234	125	71	96	186	733	1445	919
Collection rate, %*	56.93	46.47	40.11	45.93	45.93	68.38	56.82	62.22

*Number of isolates x 100/Number of cases

Appendix Table 3. Distribution of superantigen gene profiles (*speA* and *speC*) among *emm1* lineages ($M1_{UK}$, $M1_{13SNPs}$, $M1_{global}$ in STSS isolates, Japan, 2023–2024

		M1 lineage*		
Superantigen	Total*	UK	13SNPs	Global
speA	283	202	26	55
speC	8	0	7	2
speA + *speC*	175	31	143	1
Negative	4	1	0	3
Total	470	234	176	61

*Number of isolates.

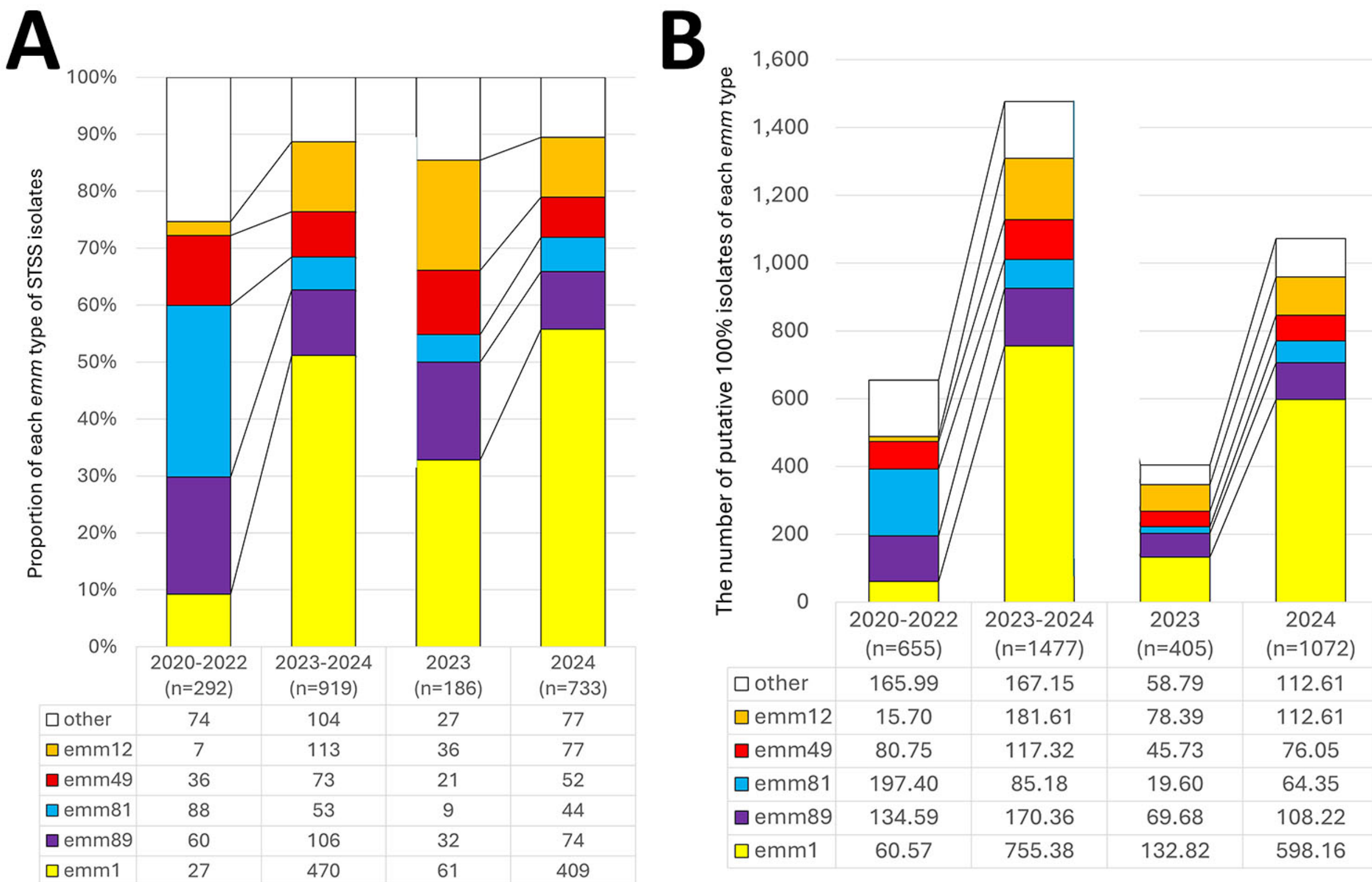

Appendix Figure 1. Prevalence of *emm* types of isolates from patients with STSS during each study year. A) Proportion of *emm* types of strains isolated from patients with STSS in 2020–2022, 2023–2024, 2023, and 2024. N indicates the number of isolates collected each year. B) Number of isolates of each *emm* type, assuming a 100% collection rate for strains during each period. N indicates the number of 100% isolates in each year.

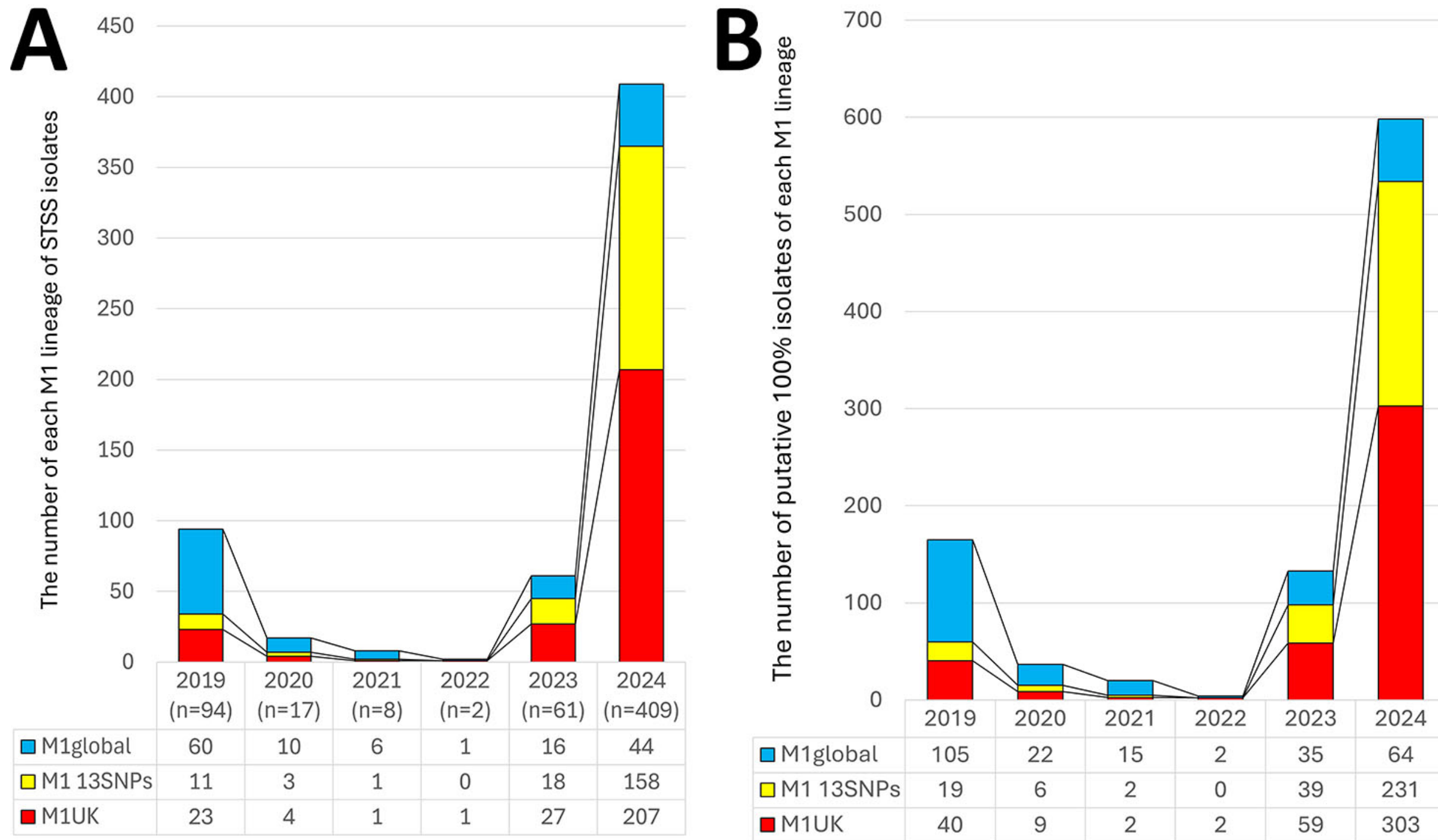

Appendix Figure 2. Prevalent of M1 lineage of isolates from patients with STSS during each study year. A) The number of *emm1* lineage of strains isolated from patients with STSS in 2019, 2020, 2021, 2022, 2023, and 2024. (B) Number of isolates of each M1 lineage, assuming a 100% collection rate for strains during each period. N indicates the number of 100% isolates in each year.
